# Water Molecules’ and Lithium Cations’ Mobility in Sulfonated Polystyrene Studied by Nuclear Magnetic Resonance

**DOI:** 10.3390/membranes13080725

**Published:** 2023-08-10

**Authors:** Stepan A. Bilyk, Vladimir A. Tverskoy, Alexander V. Chernyak, Irina A. Avilova, Nikita A. Slesarenko, Vitaly I. Volkov

**Affiliations:** 1 Federal Research Center of Problems of Chemical Physics and Medicinal Chemistry RAS, 142432 Chernogolovka, Russia; sn.bk@inbox.ru (S.A.B.); chernyak@icp.ac.ru (A.V.C.); irkaavka@gmail.com (I.A.A.); wownik007@mail.ru (N.A.S.); 2 Faculty of Fundamental Physical and Chemical Engineering, Moscow State University, 119991 Moscow, Russia; 3 Lomonosov Institute of Fine Chemical Technologies, MIREA—Russian Technological University, 119454 Moscow, Russia; vladimirtvkrskoj@gmail.com; 4 Scientific Center in Chernogolovka, Institute of Solid State Physics Named Yu. A. Osipyan RAS, 142432 Chernogolovka, Russia

**Keywords:** poly(4-styrenesulfonic acid), lithium salts, sodium salts, cesium salts, cation exchangers based on polystyrenesulfonic acid, pulsed-field gradient NMR, NMR relaxation, self-diffusion coefficient, correlation time

## Abstract

The hydration of ions and charge groups controls electro mass transfer through ion exchange systems. The self-diffusion and local mobility of water molecules as well as lithium cations in poly (4-styrenesulfonic acid) and its lithium, sodium and cesium salts were investigated for the first time using pulsed-field gradient NMR (PFG NMR) and NMR relaxation techniques. The temperature dependences of the water molecule and Li^+^ cation self-diffusion coefficients exhibited increasing self-diffusion activation energy in temperature regions below 0 °C, which is not due to the freezing of parts of the water. The self-diffusion coefficients of water molecules and lithium cations, as measured using PFG NMR, are in good agreement with the self-diffusion coefficients calculated based on Einstein’s equation using correlation times obtained from spin-lattice relaxation data. It was shown that macroscopic water molecules’ and lithium cations’ transfer is controlled by local particles jumping between neighboring sulfonated groups. These results are similar to the behavior of water and cations in sulfonic cation exchanger membranes and resins. It was concluded that polystyrenesulfonic acid is appropriate model of the ionogenic part of membranes based on this polymer.

## 1. Introduction

Recently, the number of publications on the study of electron mass transfer in ion-exchange membranes designed to create fuel cells and other electrochemical devices has increased. The majority of work relates to the measurement of ionic conductivity. Nafion membranes and their modifications, aimed at creating membranes with high proton conductivity at low moisture content, as well as cation exchange membranes based on sulfonated polystyrene, which are used in electrodialysis processes, are the most intensively studied [1,2]. NMR methods provide a unique opportunity to obtain detailed information about the state of molecules and ions, and about local molecular and ionic mobility and self-diffusion in spatial scales from tenths of nm to tens of microns, which, in turn, makes it possible to study the morphology of transport channels. The advantages of NMR also include the possibility of studying the same sample, under conditions close to native conditions, using several methods at once, which makes it possible to compare the results of various measurements and interpret them unambiguously.

Of particular interest in the study of the mechanisms of ionic conduction in the systems under consideration is the NMR with a pulsed magnetic field gradient (PFG NMR), which is a direct method for measuring the self-diffusion coefficients of water molecules and mobile ions.

In the last decade, a number of works have appeared regarding the ionic conductivity and self-diffusion of water molecules, hydrated H^+^ cations, alkali metal ions Li^+^, Na^+^, Cs^+^ in Nafion membranes [3,4,5,6,7,8,9,10,11,12,13,14,15], membranes on the basis of polyethylene with grafted sulfonated polystyrene (MSC) [8,15], poly(vinylidene fluoride)-graft-polystyrene sulfonic acid polymer electrolyte membranes [16], poly(vinyl alcohol)–poly(styrene sulfonic acid) blend membranes [17], ethylenetetrafluoroethylene-grafted-poly(styrene sulfonic) acid membranes [18], chlorosulfonated polyethylene, sulfonated polysulfone membranes [19], radiation-grafted fluoropolymers with similar poly(styrene sulfonic acid) [20], and block copolymer membranes containing sulfonated polystyrene blocks [21].

Using NMR and impedance spectroscopy, the main features of the hydration of sulfo groups, the translational mobility of water molecules, alkali metal cations, and ionic conductivity in sulfocation exchange membranes of MSC and Nafion 117 were revealed. It has been established that at low moisture contents in MSC and Nafion membranes, the H^+^ counterion forms the hydronium ion H_5_O_2_^+^, and with an increase in moisture content, H_9_O_4_^+^ is formed. The hydration numbers *h* of Li^+^, Na^+^, and Cs^+^ cations, as measured by high-resolution ^1^H NMR of water molecules, in MSC membranes are 4.1 ± 1.0, 5.0 ± 1.0, 3.1 ± 1.0, respectively, and coincide with the *h* in aqueous solutions of salt chlorides. in Nafion membranes, the *h* values are 5.0 ± 1.0, 6.0 ± 1.0, 1.0 ± 0.2. The self-diffusion coefficients of water molecules and Li^+^, Na^+^, and Cs^+^ counterions have been measured via PFG NMR on ^1^H, ^7^Li, ^23^Na, and ^133^Cs nuclei. In MSC membranes at the maximum moisture content (*RH* = 98%), the self-diffusion coefficients of cations increase in the order Li^+^ < Na^+^ < Cs^+^. The activation energies of cations’ and water molecules’ self-diffusion in the temperature range from 20 °C to 80 °C, and are close to each other (16–18 kJ/mol). The self-diffusion coefficients in Nafion membranes are arranged in the sequence Li^+^ ≥ Na^+^ > Cs^+^ [8,15]. This change in the translational mobility of cesium cations is explained by the fact that, in contrast to MSC membranes, in the Nafion membrane, the Cs^+^ cation and sulfo groups form contact ion pairs, which in particular are evidenced by the lower number of hydrated cations and the higher self-diffusion activation energy in Nafions. An NMR study of the relaxation of the ^7^Li, ^23^Na, and ^133^Cs nuclei showed that the small-scale mobility of the Li^+^, Na^+^, and Cs^+^ cations is in the same sequence as their self-diffusion coefficients [8,15]. In Nafion membranes, macroscopic cation transport is controlled by elementary jumps of hydrated cations. This may be explained by the formation of regular ionogenic channels in Nafions. The structure of membranes based on sulfonated polystyrene is inhomogeneous, as usual. These membranes’ transport pathway contains hydrated pores connected through narrow channels, and macroscopic transfer is limited by particles passing through these channels [15]. Li^+^ and Na^+^ cations have high hydration energy. Their movement is due to the rearrangement of hydrogen bonds between the water molecules of the first hydration sphere and the water molecules of the next hydration shells. The movement of the Cs^+^ cation is carried out by jumps between neighboring sulfo groups. The relationship between the structure of the polymer matrix of membranes, the hydration of cations, and the transfer of cations and water molecules on different spatial scales has been established. These factors determine the mechanisms of ionic conduction. It has been shown, in particular, that by varying the moisture content of the membranes, it is possible to create favorable conditions for the preferential transfer of the target cation.

For the interpretation of data in membranes, comparison with the results of studies of low-molecular aqueous solutions of metal chlorides turned out to be very fruitful. Obviously, for a deeper understanding of the mechanisms of ion transport in membranes and the formation of transport channels, it is necessary to study aqueous solutions of electrolytes, which are non-crosslinked polymer analogs of membranes. This is especially important, since it is the polymer chains containing sulfonic groups that form the transport channels of membranes. Such investigations can be carried out on polystyrene sulfonic acids and their salts, using different molecular weights. These studies have not yet been carried out, although NMR relaxation measurements of ^7^Li^+^ and ^23^Na^+^ cations’ nuclei in aqueous solutions of sulfonated polystyrene salts [22,23] and sulfonic cation exchangers [24] are established in the literature. The self-diffusion coefficients of polyanions were measured via PFG NMR [25]. Therefore, the application of the aforementioned techniques is a pressing task. Usually, only one technique is applied, despite a wide set of NMR techniques (NMR relaxation, PFG NMR, NMR spectroscopy) being much more informative. In order to understand the detailed mechanisms of water and cation transport, the interconnectivity of hydration, water, and cation mobility has to be revealed. It is important to compare cations with different hydration energies, Li^+^ and Cs^+^, for example. These cations are also very convenient, because ^7^Li and ^133^Cs nuclei possess rather large magnetic moments. Films of poly(4-styrenesulfonic acid) and its Li^+^, Na^+^, and Cs^+^ salts were investigated; in our opinion, these are good models of the ionogenic parts of sulfonated cation exchange membranes. Self-diffusion coefficients and the local mobility of water molecules and lithium cations were measured using pulsed-field gradient NMR and NMR spin relaxation techniques, respectively.

## 2. Materials and Methods

### 2.1. Materials

**Figure 1:**
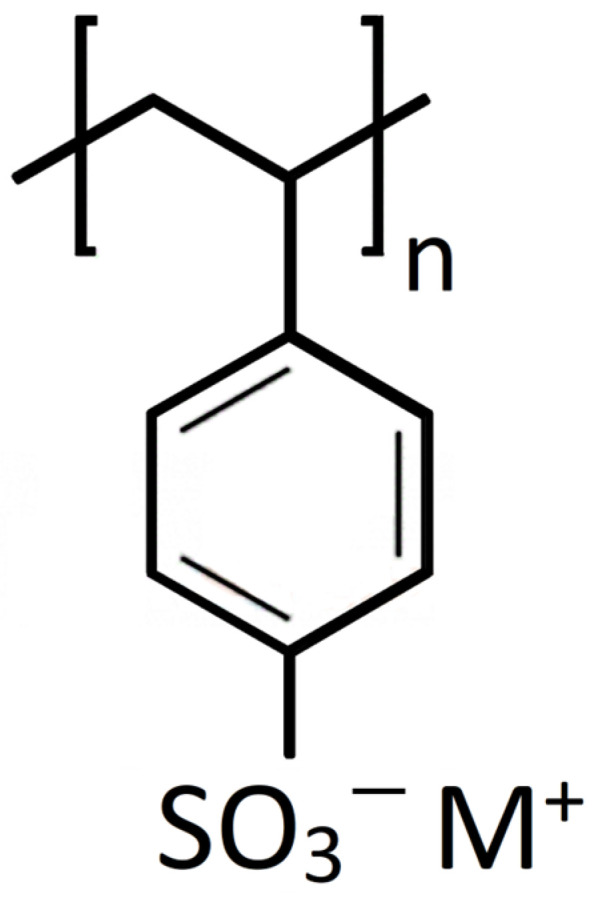
Poly(4-styrenesulfonic acid) and its lithium, sodium, and cesium salts. M^+^ is H^+^, Li^+^, Na^+^, Cs^+^.

Poly(4-styrenesulfonic acid) 18 wt % solution in H_2_O (Sigma-Aldrich, Saint Louis, MO, USA), M_w_ ~ 7.5 × 10^4^ and its lithium, sodium, and cesium salts were investigated (Figure 1).

An ^1^H NMR analysis showed that the polymer was significantly contaminated with low molecular weight fractions and impurities. Therefore, we decided to clean it up during the preparation stage. The dialysis method was applied; this consists of using a special porous membrane that allows the molecules of a substance to pass up to a certain molecular weight. A highly diluted (0.5 wt %) solution of poly(4-styrenesulfonic acid) in water was prepared, placed in a dialysis bag (Spectra/Por1 6–8 kD, 23 mm), and washed with distilled water. The special system was organized for supplying clean water and ensuring its constant flow, which increased the efficiency of the cleaning process.

**Figure 2:**
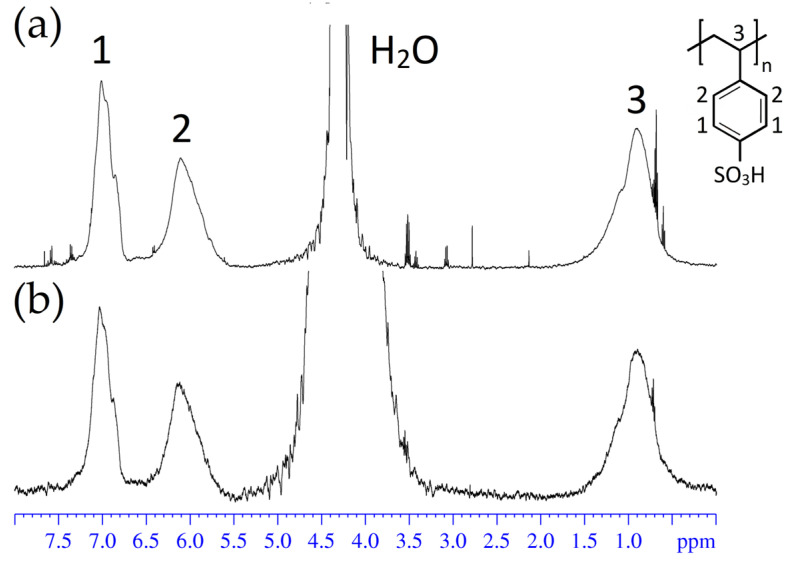
^1^H NMR spectra of polystyrene sulfonated acid aqueous solution: initial (**a**); purified (**b**). 1 and 2 are the hydrogen atoms of CH-group, 3 represents the hydrogen atoms of the CH_3_-group.

The purified solution of the substance was analyzed using high-resolution ^1^H NMR. In Figure 2, two ^1^H NMR spectra are shown; the first is the spectrum of the contaminated sulfonated polystyrene acid solution, and the second is the spectrum of the purified solution. The obtained data were compared with those for the crude solution. It was concluded that the procedure was successful.

The next step was to convert the previously purified solution of poly(4-styrenesulfonic acid) into the required ionic forms. For this purpose, NaOH, CsOH, and Li_2_CO_3_ aqueous solutions were prepared; their titers were determined via direct titration with a 1N HCl solution.

Then, a theoretically estimated two-fold excess of moles of a titrated NaOH solution was added to three samples of the polymer solution, and the average concentration of poly(4-styrenesulfonic acid) was determined via back-titration with a 1N HCl solution. Using the obtained concentration values, the available polymer solution was divided into four parts, to three of which NaOH, CsOH, and Li_2_CO_3_ solutions were added in equimolar amounts.

The polymer solutions were cast into films on a polyethylene substrate, which were crushed to the maximum possible uniformity with scissors and dried for 4 h at 80 °C in an oven to achieve a constant weight. The samples were transferred to a weighing bottle and placed in desiccators with MgCl_2_ and NaCl saturated solutions (with relative humidities of 33% and 75%, respectively), where they were kept until a constant weight was attained, after which the moistened powders were placed in NMR sample tubes.

### 2.2. Methods

NMR measurements were carried out in temperature range from −20 °C to +50 °C on Bruker spectrometers AVANCE-III-400 and AVANCE-III-500.

#### 2.2.1. Pulsed-Field Gradient NMR (PFG NMR)

**Figure 3:**
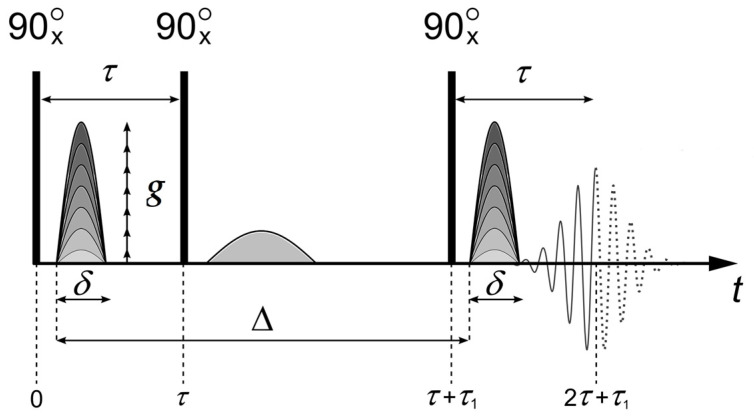
Stimulated echo pulse sequence with magnetic field gradient pulses. Here, *τ* is the time interval between the first and the second RF pulses, and *τ*_1_ is the time interval between the second and the third pulses. Δ is the interval between the gradient pulses, *δ* is duration of the equivalent rectangular magnetic field gradient pulses, *g* is the amplitude of the magnetic field gradient pulse, and *g*_0_ is the amplitude of the constant magnetic field gradient. Reprinted with permission from Ref. [13]. Copyright © 2019, Springer-Verlag GmbH Austria, part of Springer Nature.

Self-diffusion coefficients were measured for ^1^H and ^7^Li nuclei using a pulsed-field gradient technique at frequencies 400.22 and 155.51 MHz, respectively. Unfortunately, the spin–spin relaxation time T_2_ of ^23^Na and ^133^Cs was too short to apply the PFG NMR technique. The measurements were carried out on a Bruker AVANCE-III-400 NMR spectrometer equipped with a diff60 gradient unit. A pulsed-field gradient-stimulated echo sequence was used (Figure 3).

Three 90° pulses produce a stimulated spin-echo at time 2*τ* + *τ*_1_ (where *τ* is the time interval between the first and second 90° pulses, and *τ*_1_ is the interval between the second and the third pulses). The magnetic field gradient pulses of amplitude *g* and duration *δ* were applied after the first and third 90° pulses. The gradient strength was varied linearly in 32 steps within a range from 0.1 to 25.0 T/m value. Integrated intensities of spectrum lines were used to obtain the dependence of echo signal attenuation *A*(*g*) on *g*^2^ (diffusion decay) [8], according to Equation (1):

(1) $$A\left(2\tau ,\tau_{1},g\right)=A\left(2\tau ,\tau_{1},0\right)\exp\;(-\gamma^{2}g^{2}\delta^{2}t_{d}D_{s}),$$

where *γ* is the gyromagnetic ratio, Δ is the interval between gradient pulses, *t_d_* = Δ − *δ*/3 is the diffusion time, *D_s_* is the self-diffusion coefficient, and *A*(2*τ*, *τ*_1_, 0) is expressed by the following equation:

(2) $$A\left(2\tau ,\tau_{1},g\right)=\frac{A(0)}{2\exp\;(-\frac{2\tau }{T_{2}}-\frac{\tau_{1}}{T_{1}}),}$$

where *A*(0) is the signal intensity after the first radio frequency (RF) pulse. *T*_1_ and *T*_2_ are the spin–lattice and spin–spin relaxation times, respectively. During the measurement of echo signal evolution, *τ* and *τ*_1_ are fixed, and only the dependence of *A* on *g* (diffusion decay) is analyzed.

**Figure 4:**
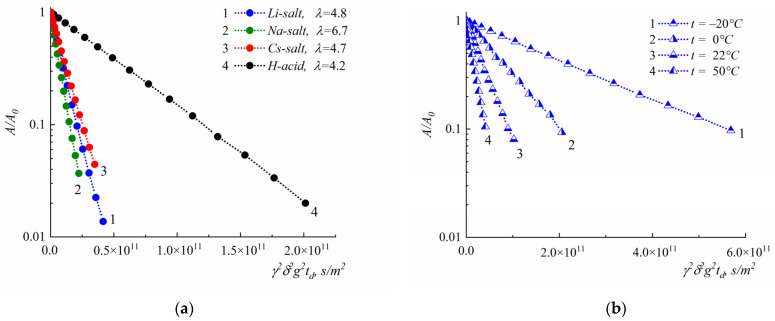
Spin echo attenuation (diffusion decay): ^1^H of water molecules in sulfonated polystyrene acid (1), lithium salt (2), sodium salt (3), and cesium salt (4) (**a**); ^7^Li of lithium cation at different temperatures (as indicated in the insertion) (**b**). For sulfonated polystyrene acid, ^1^H diffusion decay characterizes the average diffusion coefficient of hydrated H^+^ and water molecules.

Experimental diffusion decays, examples of which are shown in Figure 4, are well approximated by Equation (1), in 2–3 orders of magnitude; the self-diffusion coefficient measurement error was less than 10%.

#### 2.2.2. Spin Relaxation

Spin–lattice *T*_1_ and spin–spin *T*_2_ nuclear relaxation times were measured using 180° − *τ* − 90° and Curr–Purcel–Meighum–Gill *(*90° − *τ* − *n*180°*)* pulsed sequences, correspondingly. Longitudinal magnetization *M_z_* recovery and perpendicular magnetization *M_x_*_·*y*_ decay were approximated using exponential dependences (2) and (3), correspondingly, for the ^1^H and ^7^Li nuclei.

(3) $$\frac{\left(M_{0}-M_{z}\right)}{2M_{0}}=\exp\;(-\frac{t}{T_{1}}),$$

(4) $$M_{x}(t)=M_{0}\exp\;(-\frac{t}{T_{2}}),$$

where *M*_0_ is the equilibrium nuclear magnetization.

The spin relaxation times’ measurement error was less than 15%.

## 3. Results

### 3.1. High-Resolution NMR Spectra

**Figure 5:**
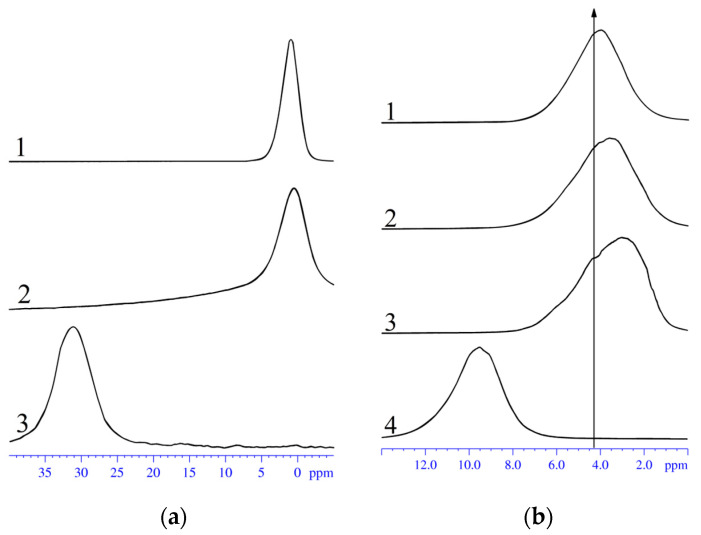
NMR spectra of ^133^Cs, ^23^Na, and ^7^Li in films of sulfonated polystyrene lithium (1), sodium (2), and cesium (3) salts (**a**). ^1^H NMR spectra of water molecules in sulfonated polystyrene (1), lithium salt, (2) sodium salt, (3) cesium salt, and (4) acid (**b**). The arrow shows the bulk water signal (4.3 ppm relative TMS).

**Figure 6:**
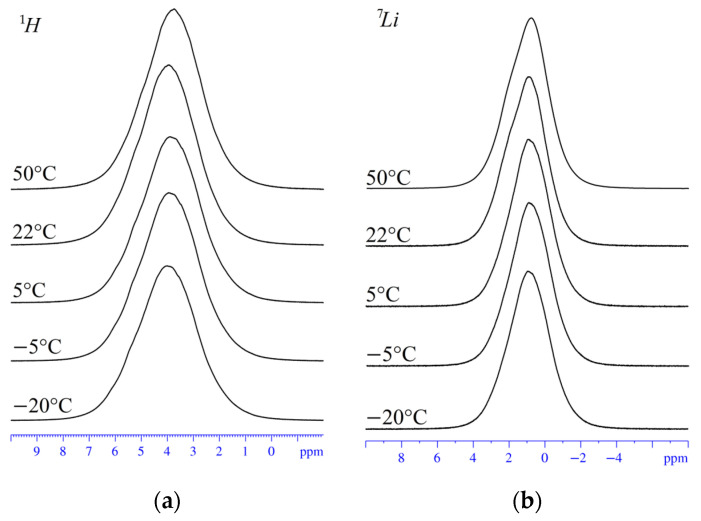
Evolution NMR spectra of ^1^H (**a**) and ^7^Li (**b**) in films of sulfonated polystyrene lithium salt with temperature variation. Temperature values are indicated in the figure.

The NMR spectra of the ^1^H and ^7^Li, ^23^Na, ^133^Cs nuclei belong to water molecules, and the Li^+^, Na^+^, Cs^+^ cations are single lines, respectively, as indicated in Figure 5a. In Figure 5b, the ^1^H NMR spectra of sulfonated polystyrene in acids and salts are shown. The hydrogen atoms of water molecules also show single lines. The ^1^H NMR spectrum in acid film features an average line of H atoms and hydrated H^+^ cations. An example of the temperature evolution of water molecules and ^1^H and Li^+^ cation spectra is shown in Figure 6; the NMR lines are rather narrow, even at temperatures below 0 °C. This indicates high water and cation mobility at low temperatures.

### 3.2. Self-Diffusion

**Figure 7:**
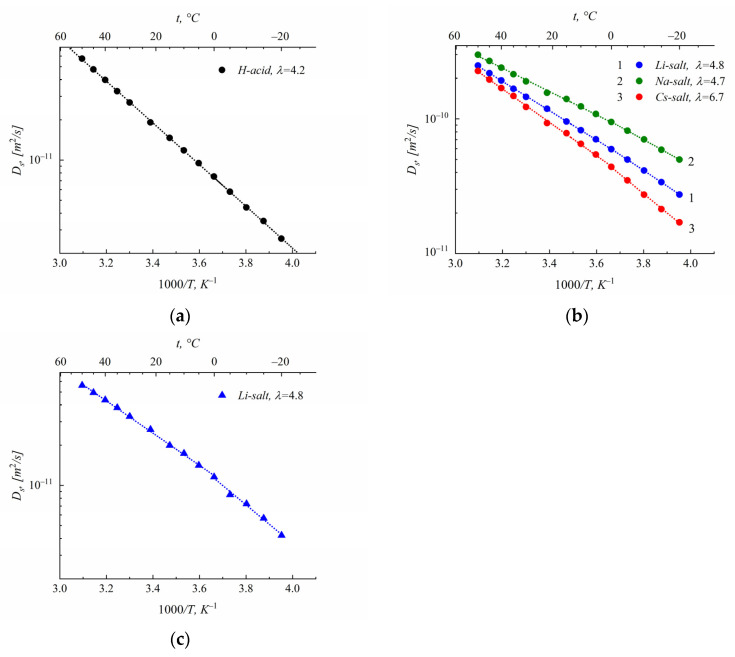
Temperature dependences of water’s self-diffusion coefficients in polystyrene acid (**a**), polystyrene lithium (1), sodium (2), and cesium (3) salts (**b**). Humidity *λ* (the number of water molecules per cation) is shown in the insertion. Temperature dependence of Li^+^ cation self-diffusion coefficient (**c**).

The temperature dependences of water’s self-diffusion coefficients are shown in Figure 7. These dependences are approximated using the Arrhenius equation:

(5) $$D=D_{0}\exp\;(-\frac{E_{a}}{RT}),$$

where *D*_0_ is the temperature-independent preexponential factor, *R* is the gas constant, *T* is the absolute temperature, and *Ea* is the self-diffusion activation energy.

The activation energies at different water contents *λ* and in low- and high-temperature regions are summarized in Table 1.

For salts’ ionic forms, activation energies increase in regions with a temperature below 0 °C (Figure 7b,c). Increases in activation energy in low-temperature regions are also observed for Li^+^ cations. The temperature dependence of ^1^H self-diffusion coefficients in polystyrenesulfonic acid at *λ* = 4.2 has the same activation shape at low and high temperatures, and the self-diffusion activation energy is 30 kJ/mole. With polystyrenesulfonic salts, water’s self-diffusion coefficients show temperature dependences on two Arrhenius curve pieces. The activation energy is about 20–30 kJ/mole, which is comparable with hydrogen bond energy. The activation energy of Li^+^ cations is a little more than that of water molecules.

The self-diffusion coefficient temperature dependences of water molecules and lithium cations *D* (*T*) in sulfonated polystyrene are typical of sulfonic cation exchangers. The approximation *D* (*T*) using two Arrhenius parts with higher activation energies in low-temperature regions was applied for different ionic forms of membranes based on sulfonated polystyrene [8] and Nafion [15], as examples. Traditionally, this increase in activation energy can be explained by the membrane water freezing, because membranes’ cooling or heating is accompanied by a peak on a DSC thermogram near 0 °C [26,27]. Usually, this peak implies the existence of two types of water molecules: “bound” and “unbound”. Bound water is immobilized; its hydrogen bonds are occupied by an interaction with sulfonated groups and cations. These water molecules are not able to form an ice phase; therefore, their mobility does not decrease at temperatures below 0 °C. Unbound (or free water) forms ice at freezing temperatures. From this point of view, the freezing part of water is the reason that the activation energy increases, because it has the same effect as decreasing humidity, which is accompanied by an increase in the activation energy [8,26,27]. At low water humidity, all water molecules are bound. In this case, the hydrogen bond structure is the same in both high- and low-temperature ranges, and no obvious DSC peak should be observed during sample cooling or heating. On the face of it, this conception is completely explained our self-diffusion data. The higher activation energy in the low-temperature region is explained by freezing of the so-called “free” water in the membrane. On the other hand, DSC peaks have also been observed in dry Nafion membranes, wherein all water molecules are bound [8,14]. In our opinion, the DSC peak does not only originate from ice; it may appear when additional hydrogen bonds are formed or destroyed.

**Figure 8:**
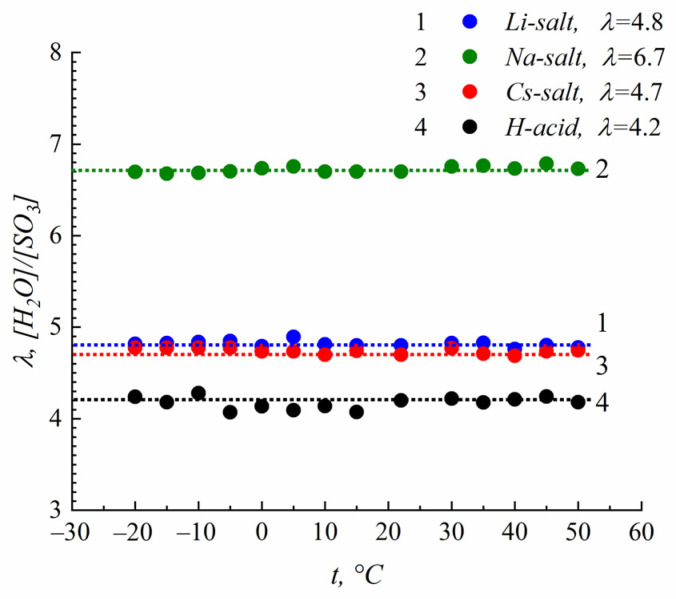
The dependence of temperature on the amount of mobile water in acid and salt ionic forms of sulfonated polystyrene.

Mobile water is easily observed using ^1^H high-resolution NMR as a rather narrow single line, as shown in Figure 5 and Figure 6. The NMR signal of water in ice is a very wide Pake doublet, which can not be fixed via a high-resolution experiment. Therefore, the formation of an ice phase follows a decrease in mobile water signal to below 0 °C. In Figure 8, the temperature dependences of mobile water in the samples indicated in Table 1 are shown. The amount of mobile water below 0 °C does not decrease, which is evidence that the water in sulfonated polystyrene films does not form an ice phase at low temperatures, despite an increase in the activation energy. This phenomenon is well known for Nafion membranes and MSC membranes based on polyethylene and grafted polystyrene at low humidity [6,8,14].

The following explanation was given for the agreement between the DSC and high-resolution NMR results. Below 0 °C, water molecules form associates, and some additional hydrogen bonds appear. These associates are still mobile, but their self-diffusion activation energy is greater than that of water molecules in high-temperature regions. At a low *λ,* water is immobilized because of interaction with cations and sulfonate groups; therefore, hydrogen bonds have the same structure in high- and low-temperature regions. This phenomenon occurs in acid polystyrene films, as shown in Figure 7a. The temperature dependence of the self-diffusion coefficient shows no curvature.

Hereby, the self-diffusion behavior of water in sulfonated polystyrene films and in sulfonic cation exchange membranes is very similar. The temperature dependence of the water self-diffusion coefficients shows an increase in activation energy in regions with a temperature below 0 °C, which is not due to the freezing of water in films.

### 3.3. Water and Lithium Cation Local Mobility

In order to investigate local water molecules and Li^+^ mobility, ^1^H and ^7^Li spin-relaxation was measured.

**Figure 9:**
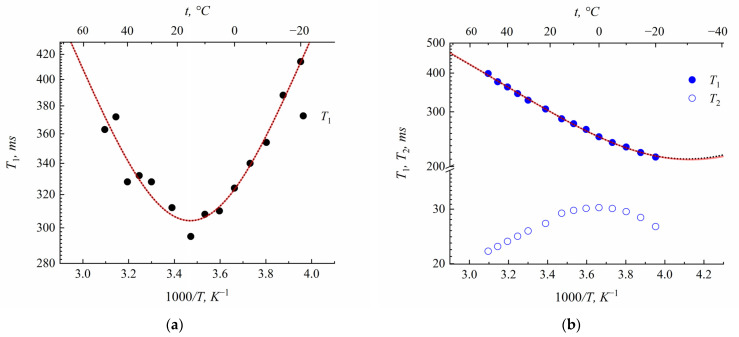

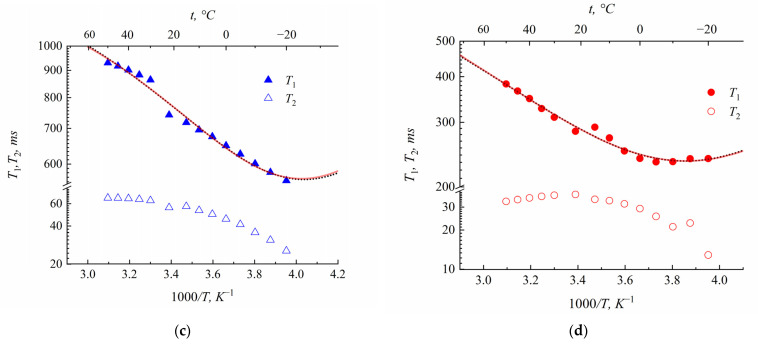
Temperature dependences of ^1^H and ^7^Li spin relaxation times: (**a**) Temperature dependence of ^1^H nuclei spin–lattice relaxation time *T*_1_ in polystyrene acid film at *RH* = 33% (*λ* = 4.2). Dotted and solid lines are Lorentz and Gauss function approximations, accordingly. (**b**) Temperature dependence of ^1^H nuclei spin–lattice relaxation time *T*_1_ and spin–spin relaxation time *T*_2_ in polystyrene lithium salt films at *RH* = 75% (*λ* = 4.8). Dotted and solid lines are Lorentz and Gauss function approximations, accordingly. (**c**) Temperature dependence of ^7^Li nuclei spin–lattice relaxation time *T*_1_ and spin–spin relaxation time *T*_2_ in polystyrene lithium salt films at *RH* = 75% (*λ* = 4.8). Dotted and solid lines are Lorentz and Gauss function approximations, accordingly. (**d**) Temperature dependence of ^1^H nuclei spin–lattice relaxation time *T*_1_ and spin–spin relaxation time *T*_2_ in polystyrene cesium salt films at *RH* = 75% (*λ* = 4.7). Dotted and solid lines are Lorentz and Gauss function approximations, accordingly.

The emperature dependences of the spin–lattice *T*_1_ and spin–spin relaxation *T*_2_ times are shown in Figure 9.

#### 3.3.1. Water Molecules’ Local Mobility and ^1^H Spin Relaxation

The spin of ^1^H nuclei is 1/2; therefore, spin relaxation occurs due to proton magnetic dipole–dipole interaction, modulated by water molecule mobility. In the case of an exponential correlation function, the dependences of relaxation rates (1/*T*_1_, 1/*T*_2_) on correlation times can be described by Bloembergen–Purcell–Pound (BPP) equations:

(6) $$\frac{1}{T_{1}}=\frac{2}{3}\gamma^{2}\langle ∆H^{2}\rangle (\frac{\tau_{c}}{1+{\left(\omega \tau_{c}\right)}^{2}}+\frac{{4\tau }_{c}}{1+{\left(\omega \tau_{c}\right)}^{2}}),$$

(7) $$\frac{1}{T_{2}}=\frac{1}{3}\gamma^{2}\langle ∆H^{2}\rangle (3\tau_{c}+\frac{{5\tau }_{c}}{1+{\left(\omega \tau_{c}\right)}^{2}}+\frac{{2\tau }_{c}}{1+{\left(2\omega \tau_{c}\right)}^{2}}),$$

(8) $$\langle ∆H^{2}\rangle =\frac{9\gamma^{2}\hbar^{2}}{20r^{6}},$$

(9) $$\tau_{c}=\tau_{c0}\exp\;(-\frac{E_{c}}{RT}),$$

where *γ* is the proton gyromagnetic ratio, *r* is the proton–proton distance, *ω* is the proton NMR frequency, and *τ_c_* is the correlation time.

Equations (6)–(9) were applied for the calculation of the correlation times *τ_c_.* In this case, the dependence of *T*_1_ on 1*/T* may be approximated using Lorentz or Gauss functions, and shows a minimum under condition *ωτ_c_* = 0.62. In the case of one correlation time at this temperature, *T*_1_*/T*_2_ equaled 1.6. As is shown in Figure 9a,b,d, the *T*_1_ (1/*T*) dependences are Lorentz- or Gaussian-shaped, but *T*_1_/*T*_2_ > 1.6. This is indicated by the correlation time’s distribution. Despite of the correlation time’s distribution, the condition of the *T*_1_ minimum, *T*_1min_, is the same (*ωτ_av_* = 0.62), where *τ_av_* is the average correlation time [15]. At these temperatures, correlation times are at a minimum; *T*_1_(*T*) is 1.97∙10^−10^ s for resonance frequencies of 400 MHz. The water’s self-diffusion coefficient may be estimated using the Einstein relation [15]:

(10) $$D=\frac{l^{2}}{6\tau_{av}},$$

where *l* is the average jumping distance.

The calculated self-diffusion coefficients were compared with experimental values measured using PFG NMR at temperatures equal to that of minimum *T*_1_. In our previous paper, the lengths of the local jumping distances of water molecules and Li^+^, Cs^+^ cations for Nafion membranes were calculated [15]. We used these distances to estimate the diffusion coefficients from Equation (10). As shown, water protons in low-humidity conditions jump 0.15 nm, which is about the length of a hydrogen bond [15]. The calculated and experimental self-diffusion coefficients at temperatures equal to the temperatures at which *T*_1_ is *T*_1min_ are given in Table 2. In the last column of Table 2, the values of *D_s_*_exp_ and *D_s_* _calc_ of the Li^+^ cation at approximately the same humidity *λ* and temperature for the Nafion 117 membrane measured in the study [15] are also given for comparison.

There is good agreement between the water’s macroscopic self-diffusion coefficients measured using PFG NMR and the self-diffusion coefficients calculated from correlation times obtained through spin–relaxation, which characterize the average water molecule jumping time.

#### 3.3.2. Local Mobility of Lithium ^7^Li Cations, and Spin Relaxation

The ^7^Li nuclear spin is 3/2. For this nucleus, the main relaxation mechanism is a quadrupole mechanism of relaxation. The spin relaxation of quadrupole nuclei has been discussed in previous papers [15,22,23,28]. The longitudinal and transversal components of magnetic relaxation curves are described by Equations (11) and (12), respectively:

(11) $$\frac{\left(M_{z}-M_{0}\right)}{M\left(cos\Theta -1\right)}=\frac{1}{5}\exp\;\left(-2J_{1}t\right)+\frac{4}{5}\exp\;\;(-2J_{2}t),$$

(12) $$\frac{\left(M_{x}\right)}{MM_{0}}sin\Theta =\frac{3}{5}\exp\;\left(-(J_{0}+J_{1})t\right)+\frac{2}{5}\exp\;(-(J_{1}+J_{2})t),$$

where *ω* is the NMR frequency, *Θ* is a rotation angle of equilibrium magnetization *M*_0_ during a radio-frequency pulse, and *J*(*λω*) represents the spectral densities on the frequencies *λω* (*λ* = 0, 1, 2).

(13) $$J_{\lambda }=0.1\pi^{2}\chi^{2}J(\omega \lambda ),$$

where

(14) $$\chi =eQ\frac{eq}{h},$$

*Q* is the nuclear quadrupole moment, *eq is the* mean square value of the electric field gradient on the nucleus, and *h* is Planck’s constant.

(15) $$J\left(\lambda \omega \right)=\frac{2\tau }{\left[1+{\left(\lambda \omega \tau \right)}^{2}\right]},$$

*τ = τ*_0_*∙*exp(*E*_a_/*RT*), where *τ* is the correlation time, and *E*_a_ is the activation energy [19,23,28].

As is shown in Equation (14), quadrupole interaction is modulated by a mean square value of the electric field gradient *eq,* which is dramatically changed when a Li^+^ cation arrives or departs the SO_3_^−^ group. Therefore, the physical sense of correlation time obtained from quadrupole relaxation is the residence time of the lithium cation in the sulfonate group. The temperature dependences of the spin–lattice and spin–spin ^7^Li nuclei relaxation times in lithium salt of sulfonated polystyrene are shown in Figure 9c.

It is very important to compare cation experimental macroscopic self-diffusion coefficients with self-diffusion coefficients calculated from local jumping times. This comparison was carried out for the Li^+^ cations. The temperature dependence of the Li^+^ self-diffusion coefficient in lithium salt of sulfonated polystyrene is shown in Figure 7c. The average correlation time was calculated from the temperature dependence of the ^7^Li spin relaxation time *T*_1_(*T*), as shown in Figure 9c. The temperature dependence of the ^7^Li spin–lattice relaxation time *T*_1_ is similar to this dependence of ^1^H, but the condition of the minimum *T*_1_ is *ωτ_c_* ≈ 1. As was mentioned above, the physical manifestation of *τ_c_* is the residence time (living time) of the Li^+^ cation near the sulfonated group. As was shown earlier, the Li^+^ cations’ jumping length is about 0.15 nm [15]. Self-diffusion coefficients calculated based on ^7^Li spin relaxation and measured via PFG NMR are indicated in Table 2. There is also good agreement between the macroscopic lithium cations’ self-diffusion coefficients and the self-diffusion coefficients calculated from local mobility. Therefore, it may be concluded that macroscopic water and cation transfer are controlled by local particle jumping between neighboring sulfonated groups. A similar phenomenon was observed in Nafion membranes, wherein ionic and molecular transport is carried out through ionogenic channels of infinite length [8,15]. Therefore, sulfonated polystyrene films may be considered a model of homogeneous cation exchange membranes. Calculated and experimentally measured self-diffusion coefficients at −20 °C and *λ* = 4.0 are also given in Table 2 [15]. These values are closed to the lithium cation self-diffusion coefficients in Nafion, found at the same temperature and humidity. These values are close to those appropriate for sulfonated polystyrene films. This exact coincidence may indicate that the structure of the ionogenic channels in Nafion and the channels in sulfonated polystyrene films are similar.

Therefore, the correlation times of water molecules and lithium cations (as typical counter ions) were calculated from ^1^H and ^7^Li spin relaxation data. On the basis of these mobilities, water and Li^+^ self-diffusion coefficients were estimated, and were found to be in good agreement with experimental values. It was concluded that macroscopic water and cation transfer is controlled by local particle jumping.

## 4. Conclusions

Water molecules and lithium cations’ self-diffusion and local mobility in poly(4-styrenesulfonic acid) and its lithium, sodium, and cesium salts were investigated using pulsed-field gradient NMR (PFG NMR) and NMR relaxation techniques. The water molecules and lithium cations’ self-diffusion coefficients were obtained via ^1^H and ^7^Li PFG NMR. Spin–lattice *T*_1_ and spin–spin *T*_2_ relaxation times were measured. The temperature dependences of the water molecules’ and Li^+^ cations’ self-diffusion coefficients and correlation times (calculated from spin relaxation data) were analyzed. The water and lithium cations’ self-diffusion activation energy in regions with a temperature below 0 °C was higher than in high-temperature regions. This is not attributed to the part of the water freezing, because number of mobile water molecules does not decrease at low temperatures. The increase in activation energy below 0 °C is explained, in our opinion, by the formation of water associates. The comparison of self-diffusion coefficients measured using PFG NMR with self-diffusion coefficients calculated from correlation times has shown that macroscopic water molecules’ and lithium cations’ transfer is controlled by local particle jumping between neighboring sulfonated groups. Therefore, ionogenic transport channels form in polystyrenesulfonic films, creating a regular structure. We therefore concluded that polystyrenesulfonic polymer films are an appropriate model of ionogenic fragments of homogeneous sulfonic cation exchangers. This result, obtained for the first time, is fundamental. Further investigation of polystyrenesulfonic aqueous solutions will allow us to understand the mechanisms of membrane transport channels’ formation. Solid-state ^13^C MAS NMR is very informative when investigating sulfonated polystyrene polymer structures [29,30]. The hydration of acid H^+^ protons was revealed via ^1^H NMR spectroscopy [30]. Pulsed-field gradient NMR was applied for investigation of the diffusion, conductivity and dissociation of LiSO_3_ groups in a water–ethanol solution of poly 4-styrene sulfonic lithium salt. It was shown that this system has potential for application in lithium batteries [31].

## Figures and Tables

**Table 1:** Water and Li^+^ self-diffusion activation energies in polystyrene acid and lithium, sodium, and cesium salts films in different water contents *λ*.

Ionic Form	Nuclear	*λ*, [H_2_O]/[SO_3_^−^]	*t* > 0 °C *E*_a1_, kJ/mole	*t* < 0 °C *E*_a2_, kJ/mole
H	^1^H	4.2	30	30
Li	^1^H	4.8	21	22
	^7^Li		23	28
Na	^1^H	6.7	16	18
Cs	^1^H	4.7	24	27

**Table 2:** Water and Li^+^ self-diffusion coefficients measured in H^+^, Li^+^, Cs^+^ sulfonated polystyrene films by PFG NMR (*D_s_*_exp_) at temperatures compared to minimum *T*_1_. Calculated water and Li^+^ self-diffusion coefficients in H^+^, Li^+^, Cs^+^ sulfonated polystyrene films (*D_s_* _calc_). *λ* is the amount of water molecules per cation. *l* is the water molecule proton and Li^+^ cation jumping length calculated in [23], at different humidities. The values of *D_s_*_exp_ and *D_s_* _calc_ of Li^+^ cations measured in [15] in a Nafion 117 membrane are also indicated for comparison.

	Water in H^+^ Acid Film	Water in Li^+^ Salt Film	Water in Cs^+^ Salt Film	Li^+^ Cation in Li^+^ Salt Film	Li+ Cation in Nafion 117 Li^+^ Ionic Form [15]
*λ*, [H_2_O]/[SO_3_^−^]	4.2	4.8	4.7	4.8	4.0
Temperature of *T*_1min_(*T*), °C	+15	−20	−5	−20	−20
*D_s_* _exp_ at *T*_1min_(*T*), m^2^/s (10^−11^)	1.5	2.7	3.5	0.3	0.4
*D_s_* _calc_ at *T*_1min_(*T*), m^2^/s (10^−11^)	2.0	3.3	1.5	0.4	0.38
*l*, nm [15]	0.15	0.20	0.15	0.15	0.15
